# Perceived Community Control in Adults with Acute Low Back Pain: A Community-Based Study

**DOI:** 10.3390/ijerph21101310

**Published:** 2024-09-30

**Authors:** Flavia P. Kapos, Colleen A. Burke, Adam P. Goode

**Affiliations:** 1 Department of Orthopaedic Surgery, Duke University School of Medicine, Durham, NC 27710, USA; colleen.a.burke@duke.edu (C.A.B.); adam.goode@duke.edu (A.P.G.); 2 Duke Clinical Research Institute, Duke University School of Medicine, Durham, NC 27701, USA; 3 Department of Population Health Sciences, Duke University School of Medicine, Durham, NC 27701, USA

**Keywords:** social determinants of health, acute pain, low back pain, pain, equity, perceived community control, sleep, community-based research

## Abstract

Background: Low back pain (LBP) is the leading cause of disability for individuals and societies globally. Prior investigations have predominantly centered around biological and psychological factors. Addressing social determinants is critical for enhancing the effectiveness and equity of pain interventions. We aimed to characterize social factors, sleep, and pain among adults with acute LBP, focusing on perceived community control. Methods: A community-based sample of adults with acute LBP was recruited from two cities in North Carolina, United States, and followed up at 3 months. We used descriptive statistics to characterize social factors, sleep, and pain, overall and by levels of perceived community control. Results: In total, 110/131 enrolled participants had data on perceived community control (lower scores indicate higher control). Overall, the median perceived community control was 14 (interquartile range [IQR] = 11, 15). People with high perceived community control also had, on average, higher perceived individual control, better-perceived neighborhood walkability, lower number of sites with bothersome comorbid pain, and higher sleep quality. A higher proportion of participants with high perceived community control were of male sex, White race, and had a higher socioeconomic position. Conclusions: Community control and related constructs may be further explored in future intervention development as potentially modifiable social factors that may reduce pain burden.

## 1. Introduction

Low back pain (LBP) is a pervasive health issue recognized as the leading cause of disability for individuals and societies globally [1]. Despite extensive research, prior investigations into chronic pain predictors have predominantly centered around biological and psychological factors [2]. A notable gap exists in understanding the role of social determinants of health in shaping the experience and management of LBP. Moreover, addressing these determinants is critical for enhancing the effectiveness and equity of pain interventions. The social dimensions of LBP are an understudied core component given the biopsychosocial recognition that pain is not solely a biological and psychological phenomenon but is profoundly influenced by the social context in which it occurs. For example, minoritized race, poorer occupational conditions, lower educational attainment, higher socioeconomic deprivation, lower income, unemployment, and rural residence are associated with higher chronic LBP prevalence, pain intensity, and pain impact, including impacts on work and social life [3,4,5]. Much less is known about the determinants of acute LBP. To identify social determinants of the transition from acute to chronic pain, which is potentially important for chronic LBP prevention and early prediction, community-based epidemiological pain research is needed.

Perceived community control reflects individuals’ beliefs regarding their ability to influence the social and environmental factors in their neighborhood or local community. It encompasses the sense of empowerment and agency individuals feel in shaping their community’s conditions and outcomes. This construct reflects not only an individual’s perception of their own control over their environment but also their confidence in their community’s collective ability to address challenges together and promote collective well-being [6]. The neighborhood social environment, defined as the sociodemographic composition of the neighborhood and its residents, as well as the relationships, groups, and social processes that exist among individuals living in the neighborhood, plays a crucial role in shaping this perception [7].

Neighborhoods characterized by strong social support networks are more likely to have a heightened sense of community control, fostering engagement in health-promoting behaviors and seeking out necessary resources [8]. Conversely, individuals within disadvantaged neighborhoods report decreased perceived control and subsequently increased stress [9] and poorer health outcomes [8,10]. Studies have shown that individuals with a strong sense of community control are more resilient in the face of adversity, exhibit lower rates of chronic diseases and mental health issues, and have improved access to healthcare resources, highlighting the critical interplay between the neighborhood social environment and individual well-being [8,9,10]. Neighborhood characteristics such as social support have been linked to LBP outcomes. Individuals residing in neighborhoods with increased social support networks are found to experience reduced disability from LBP, while decreased social support is associated with an increased prevalence of LBP [3,11,12]. Similarly, social isolation is predictive of LBP disability [3,13,14]. Therefore, community cohesion and other neighborhood characteristics may play a pivotal role in LBP outcomes.

By examining perceived community control, this line of research investigates its role as a potentially modifiable social factor that may mitigate pain burden. In this study, we aim to characterize social factors, sleep, and pain among participants of a community-based pilot study of adults with acute LBP, overall and by levels of perceived community control.

## 2. Materials and Methods

### 2.1. Study Participants

Data for these analyses come from the pilot of an ongoing cohort study of adults investigating the biopsychosocial factors related to transitioning from new onset acute LBP to chronic LBP, the Biomarkers to Advance Clinical phenotypes of low back pain, which is the BACk Study pilot. Study participants were recruited from communities in and around Durham, North Carolina (NC), and Kannapolis, NC, between February 2022 and November 2022. In Kannapolis, recruitment was primarily based on a database of existing participants of the MURDOCK Study [15], a 12,526-participant community-based longitudinal cohort originally recruited from 2007 to 2013 (enrolled ~2000 participants/year), centralized in Cabarrus County, NC. Additional recruitment methods, especially for participants in the Durham area, included advertisements on social media, newspaper articles, volunteer registries (e.g., Duke Health Volunteer Registry), emails distributed through university networks, flyers posted in or around the communities, and word of mouth. The sample size was determined by the maximum number of eligible participants that could be enrolled between February and November 2022. To be eligible for the BACk Study, individuals were required to be non-pregnant adults (>18 years old, no upper age limit) with an acute LBP episode, defined as LBP that started <4 weeks prior to screening with ≥30 days without LBP before the acute onset. Exclusion criteria were current or a previous history of systemic inflammatory or autoimmune conditions (i.e., rheumatoid arthritis, spondyloarthropathies, irritable bowel syndrome, multiple sclerosis, Guillain–Barré Syndrome, human immunodeficiency virus, or lupus), non-skin cancer, lumbar spine surgery, low back trauma, and congenital or acquired spinal defects (e.g., scoliosis). The BACk Study was reviewed and approved by the institutional review board at the Duke University School of Medicine (Pro00108542, approved 28 September 2021).

### 2.2. Measures: Social Factors

Perceived community control [6] consisted of 5 items, with total possible scores ranging from 9 to 25, which we categorized (using natural breaks in the data) into low (≥15), medium (10–14), and high perceived community control (5–9), where higher scores reflected lower perceived community control. Perceived individual control consisted of 2 items, with total possible scores ranging from 2 to 10, where higher scores reflected lower perceived individual control. Perceived opportunities for neighborhood physical activity (referred to as walkability in this manuscript) consisted of 11 items [16] (e.g., “It is pleasant to walk in my neighborhood”, “There are stores within walking distance of my home”, and “I often see other people walking in my neighborhood”), with total possible scores ranging from 11 to 55. Negative items (e.g., “My neighborhood has heavy traffic”) were reverse-coded so that increasing scores reflected worse walkability. Each item about perceived community control, perceived individual control, and perceived neighborhood walkability was scored 1–5 ranging from 1 = strongly agree, to 3 = neutral (neither agree nor disagree, and to 5 = strongly disagree [6,16].

Sociodemographic groups including age (years), sex assigned at birth (female/male), gender identity (cisgender, transgender, non-binary, genderqueer, agender, or gender fluid), and ethnicity (Hispanic/non-Hispanic) were collected via self-report [17]. Additionally, for self-reported race, participants indicated one or more identities from the following categories: American Indian or Alaska Native, Asian, Black or African American, Native Hawaiian or Other Pacific Islander, or White. Participants could also select Other and provide a write-in racial category or select Unknown or Choose Not to Respond.

Socioeconomic position was measured as educational attainment, insurance type, and financial well-being [18]. For educational attainment, participants were asked, “What is the highest level of education you have achieved?”, with the following response options: Less than High School, High School or GED, Trade/Technical/Vocational school (post-high school or GED), Some College but No Degree, Associate’s Degree, Bachelor’s Degree, Some Graduate School but No Degree, or Graduate Degree. Health insurance options were Medicaid, Medicare, Other Public Insurance, or Private Insurance. Financial well-being was measured with the question, “Which best describes your current financial situation?”, with the following response options: Live Comfortably, Meet Your Basic Expenses with a Little Left Over for Extra, Just Meet Your Basic Expenses, or Don’t Even Have Enough to Meet Basic Expenses.

### 2.3. Measures: Sleep

Sleep quality was measured using the global Pittsburgh Sleep Quality Index [19] (PSQI), with a cut-off of 5 points, which has been shown to have a sensitivity of 89.6% and a specificity of 86.5% to identify poor sleep relative to clinical and laboratory measures. The PSQI consisted of 19 items covering seven domains: sleep quality, sleep onset latency, sleep duration, sleep efficiency, sleep disturbances, use of sleeping medications, and daytime dysfunction, each scored from 0 to 3. The seven-domain sum yields the global PSQI score, ranging from 0 to 21 possible points, where higher scores reflect worse sleep.

### 2.4. Measures: Pain

LBP was defined using the Pain, Enjoyment, and General activities (PEG) [20] scale, which consisted of 3 items measuring Pain intensity and pain interference with Enjoyment of life and pain interference with General activities, where higher PEG scores indicate worse pain. LBP intensity was measured using a Numeric Rating Scale (NRS) (0–100), with 0 indicating “no pain at all” and 100 indicating “worst pain imaginable”. LBP interference was captured with the following question, “Since the onset of your low back pain, how often has pain limited your life or work activities? Would you say…”, with the options of “Never”, “Some Days”, “Most Days”, or “Every Day”.

Chronic LBP was determined at the 3-month follow-up if participants had pain “Most Days” or “Every Day” (vs. “Never” or “Some Days”) over the preceding 3 months [21]. Participants were also asked about bothersome comorbid pain (“not bothered at all”, “bothered a little”, or “bothered a lot”) in four sites, in addition to their acute LBP: “stomach”, “arms, legs, or joints other than your spine or back”, “headaches”, and “widespread pain or pain in most of your body”.

### 2.5. Statistical Analysis

We used descriptive statistics (median and interquartile range [IQR] for continuous variables or sample size [*n*=] and percentages [%] for categorical variables) to characterize social factors, sleep, and pain, overall and by levels of perceived community control. Internal consistency for perceived community control was calculated as Cronbach’s alpha, and the coefficient’s 95% confidence interval (CI) was obtained via bootstrapping with 10,000 replications. We conducted all analyses in Stata 14.1 (College Station, TX, USA). Individuals with missing data on perceived community control were excluded from analyses (*n* = 21/131 [16%] of all participants who contributed data to the BACk Study pilot). Additional missingness in other variables is noted in Table 1. We conducted a sensitivity analysis to compare the social factors, sleep, and pain of participants included with those excluded from these analyses.

## 3. Results

A total of 110 adults with acute LBP and complete data on perceived community control were included in these analyses. The median age was 58 years ([interquartile range, IQR] = 49, 70), and the sex assigned at birth was female for 60% of participants. The two most common self-reported races were White (75%) and Black or African American (18%). Overall, 53% reported having a Bachelor’s degree or higher educational attainment, while 53% reported living comfortably, and 46% reported having private insurance. The overall sample somewhat underrepresented racial/ethnic diversity and lower socioeconomic position (e.g., 7% with >than high school [HS]/HS diploma/General Education Development (GED) test, 8% “just meet basic expenses”, and 8% uninsured) (Table 1).

Sensitivity analyses suggested participants not included in the analysis due to missing data on perceived community control were on average younger (median = 51, IQR = 45, 61) than those included (median = 58, IRQ = 49, 70) and had a lower socioeconomic position (e.g., lower proportion with Bachelor’s degree or higher educational attainment, lower financial well-being, higher proportion uninsured or covered by Medicaid, and higher proportion minoritized race, but similar proportion of sex assigned at birth). Among those not included, the proportion with three or four bothersome comorbid pain sites was slightly higher, as was the baseline LBP intensity and the baseline frequency of LBP interference with life or work activities. Although only 16 of 21 of those not included in these analyses contributed data to the 3-month follow-up, sensitivity analyses showed that the proportion who transitioned to chronic LBP at 3 months was similar to that of those included in the analyses reported in the manuscript, while those not included showed slightly lower pain interference and better sleep quality.

About one-third of the sample developed chronic LBP at the 3-month follow-up. Overall, the median perceived community control was 14 of a maximum possible score of 25 points (interquartile range [IQR] = 11, 15), including 40 adults (36%) with low, 58 (53%) with medium, and 12 (11%) with high control. The internal consistency (Cronbach’s alpha) of perceived community control was 0.71 (95%CI 0.62, 0.78), which was adequate for this four-item scale [22], indicating that items are likely to reflect a single construct. Participants with high perceived community control had, on average, higher perceived individual control, better-perceived neighborhood walkability, a lower number of sites with bothersome comorbid pain, and higher sleep quality. A higher proportion of participants with high perceived community control were male sex and White race and had a higher socioeconomic position (Table 2). On the other hand, baseline pain intensity, pain interference, PEG scale, and the proportion transitioning to chronic LBP at 3 months were more similar across levels of perceived community control (Table 2).

## 4. Discussion

Perceived community control is a novel potential social determinant of LBP burden and impact. Our findings provide preliminary evidence of the link between high perceived community control (scores 5–9) and other social factors, sleep, and pain measures that may be protective against adverse LBP outcomes. Many of the observed findings between perceived community control and pain outcomes are consistent with the previous literature emphasizing the significance of social support networks and community cohesion in influencing health outcomes. Individuals with higher perceived community control demonstrated better pain-related outcomes in some measures, including better sleep quality and a lower number of sites with bothersome comorbid pain. This suggests that fostering a sense of empowerment and agency within communities could influence LBP outcomes.

Although perceived community control was not measured in a recent study of community-based adults with acute LBP in Germany, the authors reported that those with moderate or lower quality of life at baseline were 3.56 times (95%CI 1.00, 12.80) more likely to be classified as having “moderate/severe fluctuating pain” (vs. “mild/moderate fluctuating pain”). And those with lower educational attainment were 2.36 times (95%CI 0.67–8.35) more likely to be classified as having “moderate/severe fluctuating pain”—a wide confidence interval of this estimate may be a result of underrepresentation of lower-educated participants in that study [23]. Our results also highlight the interconnectedness of social factors with pain experiences. Participants with higher perceived community control reported better perceived individual control and perceived neighborhood walkability, which are generally associated with a supportive social and physical environment. These findings reinforce the importance of considering broader social determinants, such as neighborhood characteristics, built environment, and area socioeconomic position, when evaluating pain management strategies. This and other social determinants of pain may become targets for maximizing community strengths in the development of community-based pain equity interventions.

Despite the valuable preliminary insights gained from this study, which can inform the ongoing study’s approaches and the broader literature’s direction, several limitations should be acknowledged. First, the relatively small sample size (especially for adults with high perceived community control) rendered statistical significance less relevant in the interpretation of our findings (i.e., high risk of false negatives in the absence of statistical significance); nonetheless, initial findings such as ours provide important preliminary data needed to guide future hypothesis-testing studies of the broad social determinants of pain. Second, relatedly, it is possible that our results were biased by the relatively high proportion of missing data (16%)—this was better evaluated in a sensitivity analysis, which produced mixed findings. However, as previously stated, the individuals not included in the present analyses were less than 20% of the total participants, and we were reassured by verifying that a similar proportion of participants transitioned to chronic LBP at 3 months among those not included relative to those included in the analyses presented in the manuscript. Third, we did not see a clear dose–response relationship (i.e., monotonic change in the measured outcomes across groups with low, medium, and high perceived community control) for some of the reported findings. Although we saw that high perceived community control was somewhat consistently protective, the relationships with the evaluated measures were sometimes non-linear considering the low and medium perceived control groups—this could be due to a threshold effect (i.e., only perceived community control above a certain level produces a measurable protective effect on LBP outcomes) or because the observed results could not be replicated in a large enough sample due to chance findings. Future research, especially studies with an equity focus, should pursue alternative strategies (i.e., active outreach) to recruit a higher proportion of participants who are racially minoritized and have lower socioeconomic positions. Lastly, no data are available on perceived community control in these communities among adults *without* acute LBP, which would be a useful reference to compare our sample.

The social determinants of pain may hold promise for identifying actionable strategies to reduce the impact of pain and promote health equity beyond the individual level. Future research could also explore the use of other measures of perceived community control or related social constructs such as the collective efficacy scale [24], as well as community-level measures of social cohesion, collective efficacy, engagement, and control to provide a more comprehensive understanding of community dynamics as well as a more direct target for group-level interventions (e.g., fostering community connectivity, social capital, and collective empowerment). In general, the social determinants and consequences of pain are underexplored in research and practice. This research provides foundational empirical evidence for a better understanding of the social determinants of pain, which will allow focus on empowering communities and establishing community-based pain equity programs.

## 5. Conclusions

Community control and related constructs may be further explored as potentially modifiable social factors that may reduce pain burden.

## Figures and Tables

**Table 1:** Baseline sample characteristics, overall.

	Total (*n* = 110)
Age, Continuous (years) (Median [IQR])	58 (49, 70)
Age Categories (years)	
18–24	3 (3%)
25–34	4 (4%)
35–44	11 (10%)
45–54	22 (20%)
55–64	32 (29%)
65–74	26 (24%)
75+	12 (11%)
Missing	0
Sex Assigned at Birth	
Male	44 (40%)
Female	66 (60%)
Missing	0
Race	
White	82 (75%)
Black/African American	20 (18%)
Multiracial	5 (5%)
Asian	1 (1%)
Unknown	2 (2%)
Missing	0
Educational Attainment	
High School Graduate, GED, or Lower	7 (6%)
Some College/Trade/Technical/Associate’s Degree	45 (41%)
Bachelor’s Degree or Higher	58 (53%)
Missing	0
Financial Well-Being	
Live Comfortably	52 (53%)
Meet Basic Expenses with a Little Extra	38 (39%)
Just Meets Basic Expenses	8 (8%)
Don’t Even Have Enough to Meet Basic Expenses	0 (0%)
Missing	12
Insurance Type	
Uninsured	7 (7%)
Medicaid	1 (1%)
Medicare	33 (32%)
Other Public	13 (12%)
Private	48 (46%)
Unsure	2 (2%)
Missing	6

IQR = interquartile range; GED = General Educational Development exam, a high school equivalency credential in the United States.

**Table 2:** Sample characteristics, by level of perceived community control.

Characteristic	High Control	Medium Control	Low Control
*N*	12	58	40
Age, Continuous (Baseline, years) (Median [IQR])	58 (47, 65)	59 (51, 71)	56 (48, 70)
Sex Assigned at Birth (Baseline)			
Male	8 (67%)	21 (36%)	15 (38%)
Female	4 (33%)	37 (64%)	25 (62%)
Self-Reported Race (Baseline)			
White	10 (83%)	42 (72%)	30 (75%)
Black/African American	2 (17%)	10 (17%)	8 (20%)
Multiracial	0 (0%)	4 (7%)	1 (2%)
Asian	0 (0%)	1 (2%)	0 (0%)
Unknown	0 (0%)	1 (2%)	1 (2%)
Educational Attainment (Baseline)			
High School Graduate, GED, or Lower	0 (0%)	2 (3%)	5 (12%)
Some College/Trade/Technical/Associate’s Degree	3 (25%)	29 (50%)	13 (32%)
Bachelor’s Degree or Higher	9 (75%)	27 (47%)	22 (55%)
Financial Well-Being (Baseline)			
Live Comfortably	9 (75%)	23 (43%)	20 (61%)
Meet Basic Expenses with a Little Extra	3 (25%)	26 (49%)	9 (27%)
Just Meets Basic Expenses	0 (0%)	4 (8%)	4 (12%)
Don’t Even Have Enough to Meet Basic Expenses	0 (0%)	0 (0%)	0 (0%)
Insurance Type (Baseline)			
Uninsured	0 (0%)	3 (5%)	4 (11%)
Medicaid	0 (0%)	1 (2%)	0 (0%)
Medicare	2 (18%)	19 (34%)	12 (32%)
Other Public	2 (18%)	6 (11%)	5 (14%)
Private	7 (64%)	25 (45%)	16 (43%)
Unsure	0 (0%)	2 (4%)	0 (0%)
Pain Intensity (Baseline) (Median {IQR])	2 (2, 4)	4 (2, 5)	3 (2, 5)
Baseline Pain Interference			
Never	2 (17%)	8 (14%)	12 (30%)
Some Days	9 (75%)	45 (78%)	25 (62%)
Most Days	1 (8%)	4 (7%)	2 (5%)
Every Day	0 (0%)	1 (2%)	1 (2%)
PEG Scale (Baseline) (Median [IQR])	2 (1, 4)	3 (2, 5)	3 (1, 5)
Number of Bothersome Comorbid Pain Sites (Baseline)			
0	0 (0%)	14 (24%)	5 (12%)
1	5 (42%)	21 (36%)	11 (28%)
2	4 (33%)	13 (22%)	17 (42%)
3	3 (25%)	3 (5%)	5 (12%)
4	0 (0%)	7 (12%)	2 (5%)
Chronic LBP (3 Months)	3 (27%)	19 (36%)	11 (29%)
Perceived Neighborhood Walkability (Baseline) (Median [IQR])	22 (19, 28)	27 (23, 30)	28 (24, 33)
Global PSQI Score (Baseline)			
<5	6 (50%)	15 (26%)	11 (28%)
≥5	6 (50%)	43 (74%)	29 (72%)

IQR = interquartile range; GED = General Educational Development exam, a high school equivalency credential in the United States. PEG = Pain intensity and pain interference with Enjoyment of life and General activities; bothersome comorbid pain sites were a count of sites that “bothered little” or “bothered a lot” (vs. “not bothered at all”), including “stomach”, “arms, legs, or joints other than your spine or back”, “headaches”, and “widespread pain or pain in most of your body”. LBP = low back pain; PSQI = Pittsburgh Sleep Quality Index.

## Data Availability

The datasets presented in this article are not readily available because they are part of an ongoing study and participant confidentiality must be maintained. Requests to access the datasets should be directed to A.P.G.
